# Functional varying-coefficient Cox model and its application

**DOI:** 10.1177/09622802251406527

**Published:** 2026-01-02

**Authors:** Fansheng Kong, Maozai Tian, Zhihao Wang, Man-lai Tang

**Affiliations:** 1 Center for Applied Statistics, School of Statistics, 12471 Renmin University of China, PR China; 2 School of Statistics and Data Science, 12614 Xinjiang University of Finance and Economics, PR China; 3 Department of Physics, Astronomy and Mathematics, 3769 University of Hertfordshire, UK

**Keywords:** Functional data, varying-coefficients, Cox model

## Abstract

When data become increasingly complex, desirable models are required to be more flexible for analyzing survival data. Building upon the existing functional Cox model, we introduce a novel functional varying-coefficient Cox model and the corresponding estimation algorithms are proposed in this article. The proposed model can simultaneously handle survival data with varying-coefficient covariates and functional covariates, thereby significantly enhancing the adaptability of survival models. The model performance is evaluated by simulation studies, and a real application using Alzheimer’s disease neuroimaging initiative (ADNI) data is used to illustrate the practicality of the proposed model.

## 1. Introduction

With the development of the economy, an increasing amount of time-to-event data is being observed and recorded, and the analysis of such data is widely applied in various fields, including medicine research, financial studies, and social science investigation. In biomedical research, time-to-event data are also known as survival data, and the analysis of time-to-event data is referred to as survival analysis. Survival analysis investigates the relationship between certain characteristics of study subjects (e.g. patients) and the time until an event occurs, such as death of the study subject, failure time of certain experiments or recovery. A common model for analyzing such data in statistical research is the hazard model, which associates the risk of an event (usually the onset of a disease or death) with one or more covariates related to that event. The Cox proportional hazards model is a very common type of hazard model and a popular model for analyzing time-to-event data with censored data and covariates.^1^

An important assumption of the Cox proportional hazards model is that the hazard remains constant over time. For example, taking a specific drug may halve a person’s possibility of getting a disease, or changing the specific material of certain manufacturing components may triple the possibility of their failure. However, in real-life application, this assumption of constant hazard may not be realistic. In clinical medicine, such non-constant hazard phenomena are quite common. For example, when studying the occurrence of mild cognitive impairment (MCI) related to time to death, the presence of MCI among participants may change over time. One explanation is the existence of covariates that change over time, which are referred to as time-varying covariates. Leemis et al.^2^ proposed an algorithm for generating lifetimes when survival models include time-varying covariates, primarily based on inverting the cumulative hazard function to generate survival times. Austin^3^ studied the data generation process for the Cox proportional hazards model with time-varying covariates under exponential, Weibull, or Gompertz distributions. Yan and Huang^4^ proposed an adaptive lasso method for variable selection in Cox models with time-varying covariates. Ngwa et al.^5^ used the Lambert W function to generate survival time data for the Cox proportional hazards model with time-varying covariates that follow exponential or Weibull distributions, and demonstrated that reliable and robust estimation can be performed through simulation studies. Cygu et al.^6^ studied the penalized Cox proportional hazards model in the presence of a large number of time-varying covariates and proposed a variant of the gradient descent algorithm to fit the penalized Cox model.

Another extension of the Cox proportional model is the expansion of coefficients, transitioning from originally fixed coefficients to time-varying coefficients. In this case, it is generally assumed that the varying coefficients depend on certain covariates, such as scores for disease severity. Typically, the variation of coefficients is related to the variation of time t , known as time-varying coefficients. In such cases, it is referred to as the Cox regression model with time-dependent or time-varying coefficients. Zucker and Karr^7^ and Hastie and Tibshirani^8^ conducted initial explorations of varying coefficient models. Cai et al.^9^ developed a local partial likelihood method to estimate time-dependent coefficients in the Cox regression model and obtained the asymptotic properties of the estimators, where the time-varying functions were expanded using first-order Taylor functions. Kim et al.^10^ studied the issue of missing data in varying coefficient proportional hazards models and developed a reverse probability-weighted estimator based on the local partial likelihood method. Song et al.^11^ considered the Bayesian analysis of proportional hazards models with time-varying coefficients under two prior scenarios. Kim et al.^12^ and Yang et al.^13^ considered the estimation problem of time-varying coefficient models with left-truncated data and latent variables, respectively.

Functional data analysis (FDA) has become increasingly popular in many fields, such as medical research, magnetic resonance imaging (MRI), meteorology and public health. The most common method for modeling functional data is the functional linear model (FLM). There are many articles about the extension of functional linear models. Shin^14^ introduced the partially functional linear regression model (PFLRM). Peng et al.^15^ extended the partially functional linear model and proposed the varying coefficient partially functional linear regression model (VCPFLM) based on Shin^14^. Wang et al.^16^ proposed the functional partially varying coefficient zero-inflated model (FPVCZIM). When modeling Cox hazard models, the covariates related to the interesting event include various types, commonly binary and numerical types, which are relatively simple to model. As data complexity increases, functional data is also incorporated into survival analysis models. Modeling functional data is more complex than modeling general numerical data. Some scholars have combined functional data with Cox models for modeling. Lee et al.^17^ proposed a Bayesian functional linear Cox regression model (BFLCRM) with functional and scalar covariates. Kong et al.^18^ proposed a functional linear Cox regression model to describe the association between event occurrence time data and a set of functional and scalar predictor variables, and developed an algorithm to calculate the maximum approximate partial likelihood estimation of unknown finite and infinite-dimensional parameters. In the final real data analysis, they showed that high-dimensional hippocampal surface data may be an important marker for predicting the time to conversion to Alzheimer’s disease.

According to our knowledge, there are few articles that combined Cox hazard models with functional data, and those articles only considered modeling with functional covariates and fixed coefficients. However, in real life applications, varying coefficients are more common than fixed coefficients. In this article, we therefore propose the so-called functional varying-coefficient Cox (FVC-Cox) model, which extends the analysis of Cox hazard models to a framework that simultaneously incorporates both varying and fixed coefficients, as well as functional data. This extension can be better adapted to practical applications, and is more interpretive and flexible. In this model, functional principal components (FPCs) are used to approximate the functional covariates, B-spline methods are used to approximate the varying coefficient covariates. The performance of the proposed method is evaluated through simulation studies. A real application using Alzheimer’s disease neuroimaging initiative (ADNI) data is utilized to illustrate the practicality of the proposed model.

## 2. Functional varying-coefficient Cox (FVC-Cox) model and estimation

### 2.1. Functional varying-coefficient Cox (FVC-Cox) model

Assume that $X_{i}=(X_{i1},X_{i2},\ldots ,X_{ip}{)}^{T}$ is a p -dimensional covariate vector for individual i . The general Cox proportional hazards model proposed at time t is defined as follows:

$$h_{i}\left(t|X_{i}\right)=h_{0}(t)\exp\;\left(X_{i}^{T}\beta \right)=h_{0}(t)\exp\;\left(\beta_{1}X_{i1}+\beta_{2}X_{i2}+\cdots +\beta_{p}X_{ip}\right),$$

where $h_{0}(t)$ is the baseline hazard function, and the vector $X_{i}$ remains fixed over time within the same individual. The parameter $\beta =(\beta_{1},\ldots ,\beta_{p})$ represents the coefficients, which also remain constant.

For functional data, we assume that the response variable Y is a real-valued random variable which defined on the probability space (Ω,B,P) , {X(t):t∈T} is a second-order stochastic process defined on the same probability space with $\left\{E|X(t){|}^{2}<\infty ,\forall t\in T\right\}$ , and $X\in L^{2}(T)$ with $L^{2}(T)$ being the Hilbert space of square-integrable functions defined on a non-degenerate compact interval T . Without loss of generality, we let T be the unit interval [0,1] . Therefore, the relationship between X(t) and Y can be expressed as:

(1) $$Y=\int_{T}X(t)\beta (t)dt+\varepsilon .$$

Model (1) is commonly referred as the functional linear model (FLM), where X(t) is the functional predictor variable and β(t) is the unknown functional coefficient.

### 2.2. Functional varying-coefficient Cox model (FVC-Cox)

Kong et al. (2018) proposed the functional linear Cox regression model (FLCRM) which introduced functional data into the Cox regression model to describe the relationship between survival outcome and a set of finite-dimensional and infinite-dimensional predictors. The corresponding hazard model is defined as follows:

$$h(t|X,Z)=h_{0}(t)\exp\;\left(Z\gamma +\int_{T}X(t)\beta (t)dt\right).$$

In this model, the hazard function is related to the functional covariate X(t) and the general covariate Z with the covariate coefficient γ being fixed. This model incorporates functional covariates into the general Cox model, increasing the flexibility of the model. In practice, the covariate coefficient γ is sometimes not completely fixed. Therefore, we consider FVC-Cox that could simultaneously considers functional covariates and varying coefficients. Specifically, for the i -th individual, the hazard function for the FVC-Cox has the following form

(2) $$h_{i}(t|X_{i},Z_{i},V_{i})=h_{0}(t)\exp\;\left(V_{i}^{T}\theta +Z_{i}^{T}\alpha (U)+\int_{T}X_{i}(t)\beta (t)dt\right).$$

where covariates $V_{i}$ , $Z_{i}$ and $X_{i}(t)$ for individual i can be observed, and parameter θ is a fixed coefficient. Without loss of generality, we assume that U is a one-dimensional variable, $\alpha (U)=(\alpha_{1}(U),\alpha_{2}(U),\ldots ,\alpha_{p}(U){)}^{T}$ is a p -dimensional vector of unknown varying coefficients, and β(t) is the functional coefficient. Specifically, when the parameter α(U)=0 , Model (2) reduces to the FLCRM (i.e. Model (1)).

## 3. Estimation process

As β(t) is an infinite dimensional parameter, we need to transform the problem of infinite parameters into that of finite parameters. For this purpose, we define the covariance function of the functional predictor variable X(t) as K(s,t)=Cov(X(s),X(t)) . Assume that K(s,t) is continuous on $[0,1{]}^{2}$ .

According to Mercer’s Theorem, we have $K(s,t)=\sum_{j=1}^{\infty }\lambda_{j}\phi_{j}(s)\phi_{j}(t)$ , where $\lambda_{j}$ are the eigenvalues $\left(\lambda_{1}>\lambda_{2}>\cdots >0\right)$ and $\phi_{j}(s),j=1,2,\ldots$ are the continuous orthonormal eigenfunctions corresponding to the covariance operator of K(s,t) . Define the inner product on space T as $<f(t),g(t)>=\int_{T}f(t)g(t)dt$ . Hence, the covariance operator can be defined as: <K,f>(u)=∫K(u,v)f(v)dv , where $f\in L^{2}(T)$ . The empirical covariance function of X is defined as $\hat{K}(s,t)=\frac{1}{n}\sum_{i=1}^{n}X_{i}(s)X_{i}(t)$ . If ${\hat{\lambda }}_{1}\geq {\hat{\lambda }}_{2}\geq \cdots \geq 0$ is the ordered sequence of eigenvalues of $\hat{K}$ , the spectral decomposition of $\hat{K}$ can be written as:

$$\hat{K}(s,t)=\sum_{j=1}^{\infty }\hat{\lambda_{j}}{\hat{\phi }}_{j}(s){\hat{\phi }}_{j}(t).$$

It is noteworthy that the sequence $\phi_{j}{\left(s\right)}^{\prime }s$ also form an orthonormal basis on $[0,1{]}^{2}$ . According to the Karhunen–Loève Theorem, we have: $X(t)=\sum_{i=1}^{\infty }\xi_{i}\phi_{i}(t)$ , where $\xi_{i}^{\prime }s$ are uncorrelated random variables with zero mean and variance $\lambda_{i}$ , and $\xi_{i}=\left\langle X,\phi_{i}\right\rangle$ .^19^

Similarly, we can obtain the decomposition:

$$X_{i}(t)=\sum_{j=1}^{\infty }{\hat{\xi }}_{ij}{\hat{\phi }}_{j}(t),\beta (t)=\sum_{j=1}^{\infty }\eta_{j}{\hat{\phi }}_{j}(t),$$

where ${\hat{\xi }}_{ij}=<X_{i},{\hat{\phi }}_{j}>$ and $\eta_{j}=<\beta ,{\hat{\phi }}_{j}>$ . Substituting these decompositions into the functional part, we have:

(3) $$\begin{array}{rl} \int_{0}^{1}X_{i}(t)\beta (t)dt & =\int_{0}^{1}\sum_{j=1}^{\infty }{\hat{\xi }}_{ij}{\hat{\phi }}_{j}(t)\sum_{k=1}^{\infty }\eta_{k}{\hat{\phi }}_{k}(t)dt\\ & =\sum_{j=1}^{\infty }\sum_{k=1}^{\infty }{\hat{\xi }}_{ij}\eta_{k}\int_{0}^{1}{\hat{\phi }}_{j}(t){\hat{\phi }}_{k}(t)dt\\ & =\sum_{j=1}^{\infty }{\hat{\xi }}_{ij}\eta_{j}. \end{array}$$

Substituting Equation (3) into Equation (2) yields:

(4) $$h_{i}(t|X_{i},Z_{i},V_{i})=h_{0}(t)\exp\;\left(V_{i}^{T}\theta +Z_{i}^{T}\alpha (U)+\sum_{j=1}^{\infty }{\hat{\xi }}_{ij}\eta_{j}\right).$$

The term $\sum_{j=1}^{\infty }{\hat{\xi }}_{ij}\eta_{j}$ in Equation (4) is infinite-dimensional. Here, we approximate it by using the first m terms. That is,

(5) $$h_{i}(t|X_{i},Z_{i},V_{i})=h_{0}(t)\exp\;\left(V_{i}^{T}\theta +Z_{i}^{T}\alpha (U)+\sum_{j=1}^{m}{\hat{\xi }}_{ij}\eta_{j}\right).$$

The effectiveness of this approximation depends on whether the first m terms can closely approximate the original term. The choice of m has relatively significant impact on the estimation accuracy. In general, a smaller m leads to larger bias while a larger m may result in larger variance. The method for selecting m will be discussed later.

For the varying coefficient part, local polynomials or B-splines are commonly used for approximation. Since B-spline approximation is global and computationally efficient and fewer parameters need to be estimated, we adopt B-spline approximation for the varying coefficient part in this paper. Let $k_{n}$ be the number of uniform internal knots on [0,1] , h+1 be the order, and the corresponding B-spline basis be $\pi (u)=(B_{1}(u),B_{2}(u),\ldots ,B_{k_{n}+h+1}(u){)}^{T}$ . Thus, the varying coefficient α(U) can be approximated by

(6) $$\alpha_{i}(U)\approx \sum_{j=1}^{k_{n}+h+1}B_{j}(u)\gamma_{i,j}=\pi^{T}(u)\gamma_{i},\;i=1,2,\ldots ,p,$$

where $\gamma_{i}=(\gamma_{i,1},\ldots ,\gamma_{i,k_{n}+h+1}{)}^{T}$ is the coefficient vector of the spline basis. In this paper, we choose h=3 , that is, cubic splines. Substituting Equation (6) into Equation (5), we have

(7) $$h_{i}(t|X_{i},Z_{i},V_{i})=h_{0}(t)\exp\;\left(V_{i}^{T}\theta +\sum_{k=1}^{p}\sum_{j=1}^{k_{n}+h+1}Z_{ik}B_{j}(u)\gamma_{kj}+\sum_{g=1}^{m}{\hat{\xi }}_{jg}\eta_{g}\right).$$

We outline the estimation procedure for the parameters in the model as follows:

Step 1: Using B-splines to smooth the function α(U) for individual i . This process yields a smooth estimate of α(U) . The values of α(U) within interval T can be calculated and expressed as a combination of spline basis functions.

Step 2: Using FPC Analysis to decompose the functional sequence ${\hat{X}}_{i}(t)$ into the product of principal component scores with principal components. To achieve a better fitting result, a reasonable method to select m is necessary. This allows ${\hat{X}}_{i}(t)$ to be approximated as a linear combination.

For selecting the value of m , there are several methods. One method is to set a threshold for the first m FPC variances, denoted as $PV(m)=\sum_{i=1}^{m}\lambda_{i}/\sum_{i=1}^{\infty }\lambda_{i}$ . For example, one might set PV≥95% or PV≥90% , we can then get the corresponding value m . Another method is similar to variable selection. Since changing the value of m can significantly affect the accuracy of the estimation. Generally, a smaller m results in larger bias while a larger m may lead to larger variance. Therefore, the AIC criterion can be used to select the value of m . Here, AIC is defined as: AIC(m)=2m−2ln{L} , where L is the likelihood function.

Step 3: Substituting the approximated results from Steps 1 and 2 into the proposed FVC-Cox Model. The parameter $\hat{\delta }=({\hat{\theta }}^{T},{\hat{\alpha }}^{T},{\hat{\eta }}^{T}{)}^{T}$ can be estimated by maximizing the following log partial likelihood equation:

$$Q(\delta )=\sum_{i=1}^{n}\int_{T}{\hat{w}}_{i}^{T}\delta dN_{i}(t)-\int_{T}\log\;\left\{\sum_{i=1}^{n}Y_{i}(t)\exp\;\left({\hat{w}}_{i}^{T}\delta \right)\right\}d\bar{N}(t),$$

where $N_{i}(t)=I({\tilde{T}}_{i}\leq t,\delta_{i}=1)$ , $\bar{N}(t)=\sum_{i=1}^{n}N_{i}(t)$ , and $R(t)=\{j:{\tilde{T}}_{j}\geq t\}$ represents the set of subjects at risk and uncensored before time t . Define $Y_{i}(t)=I({\tilde{T}}_{i}\geq t)=I(i\in R(t))$ and ${\hat{w}}_{i}=({\hat{\theta }}_{i1},\ldots ,{\hat{\theta }}_{ip_{1}},\alpha_{i1},\ldots ,\alpha_{ip_{2}},\eta_{i1},\ldots ,\eta_{ip_{m}}{)}^{T}$ for i=1,…,n . In this paper, it is assumed that the observed failure times are distinct.

## 4. Large sample properties

In this section, we will study some properties of the estimators. Given (θ,α,β) , let $F_{\varepsilon }(\cdot )$ and $f_{\varepsilon }(\cdot )$ be the conditional distribution function and conditional density function of ε , respectively. When U=1 , the varying coefficients α(U) can be converted to the parameter θ . Thus, the properties of the parameter θ can be incorporated into the parameter α(U) . Let K represent a positive constant that may take different values in different contexts. $\alpha_{0}(\cdot )$ and $\beta_{0}(\cdot )=(\beta_{01}(\cdot ),\ldots ,\beta_{0p}(\cdot ){)}^{⊤}$ denote the true values of α(⋅) and β(⋅) , respectively. $a_{n}≍b_{n}$ indicates that $\frac{a_{n}}{b_{n}}$ has a lower bound greater than 0 and a finite upper bound.

We consider the following conditions (i.e. (A1)–(A5)) on the considered survival data. Here, Conditions (A1)–(A4) can be regarded as a direct extension of some standard conditions in the literature.

(A1) $\int_{0}^{\tau }h_{0}(t)dt<\infty$ .

(A2) Let $w_{iR}=(\xi_{i1R},\ldots ,\xi_{ir_{n}R},z_{i1},\ldots ,z_{ip}{)}^{T}$ . For d=0,1,2 , we define

$$S^{\left(d\right)}\left(\eta_{R},t\right)=n^{-1}\sum_{i=1}^{n}{\left(w_{iR}\right)}^{\left(d\right)}Y_{i}(t)\exp\;\left(\eta_{R}^{T}w_{iR}+e_{i}\right),$$

where $(w_{iR}{)}^{\left(0\right)}=1$ , $(w_{iR}{)}^{\left(1\right)}=w_{iR}$ and $(w_{iR}{)}^{\left(2\right)}=(w_{iR}{)}^{⨂2}$ . Moreover, there exists a neighborhood B of the true value of $\eta_{R}$ , denoted as $\eta_{R0}$ , and a scalar, a vector or a matrix continuous function $s^{\left(d\right)}(\eta_{R},t)=ES^{(}d)(\eta_{R},t)$ defined on B×[0;τ] such that

$$\underset{t\in [0,\tau ],\eta_{R}\in B}{\sup}\left\|S^{\left(d\right)}\left(\eta_{R},t\right)-s^{\left(d\right)}\left(\eta_{R},t\right)\right\|{⟶}^{p}0\;\text{for }d=0,1,2.$$

(A3) The functions $s^{\left(d\right)}$ for d=0,1,2 are bounded on B×[0;τ] and $s^{\left(d\right)}(\cdot ;t)$ are continuous in $\eta_{R}\in B$ uniformly in t∈[0;τ] . Moreover, $s^{\left(0\right)}$ is bounded away from 0 on B×[0;τ] .

(A4) The matrix $\Sigma (\eta_{R0})=\int_{0}^{\tau }v(\eta_{R0},t)s^{\left(0\right)}(\eta_{R0},t)h_{0}(t)dt$ is positive definite, where $v(\eta_{R},t)=\{s^{\left(0\right)}{\}}^{-1}s^{\left(2\right)}-\{s^{\left(0\right)}{\}}^{-2}\{s^{\left(1\right)}{\}}^{⊗2}$ .

(A5) For any 1≤k≤p , $z_{k}$ is subgaussian.

(A6) $\lambda_{j}-\lambda_{j+1}\geq Kj^{-a-1}$ for j≥1 with a>1 .

(A7) $|\eta_{j0}|\leq CK^{-b}$ for j>1 .

(A8) For all j≥1 , $E||X(\cdot )|{|}^{4}\leq K$ , and $E[\xi_{j}^{4}]\leq K\lambda_{j}^{2}$ .

(A9) For a>1 , $K^{-1}j^{-a}\leq \lambda_{j}\leq Kj^{-a}$ and for all j≥1 , the eigenvalues $v_{j}^{\prime }s$ satisfy $v_{j}-v_{j+1}\geq K^{-1}j^{-a-1}$ .

(A10) The truncation number m satisfies $m≍n^{\frac{1}{a+2b}}$ .

(A11) For all u∈[0,1] , matrix $E(ZZ^{⊤}|U=u)$ is positive definite matrix, with eigenvalues greater than 0 and uniformly bounded.

(A12) Varying coefficient $\alpha_{k}(u)(k=1,\ldots ,p)$ has continuous q -th order derivatives $\alpha_{k}^{\left(q\right)}(u)(k=1,\ldots ,p)$ , and satisfies

$$\left\|\alpha_{k}^{\left(q\right)}(u)-\alpha_{k}^{\left(q\right)}\left(u^{\prime }\right)\right\|\leq K{\left|u-u^{\prime }\right|}^{v},\;0\leq u,u^{\prime }\leq 1,\;k=1,\ldots ,p,$$

where K>0,0<v≤1 , and r=q+v,r≥1 .

(A13) The number of knots $k_{n}$ in the space $S_{n}$ composed of spline functions satisfies $k_{n}≍n^{\frac{1}{1+2r}}$ .

(A14) For l=1,…,p , $\alpha_{0l}(\cdot )$ is r -th ( r≥2 ) continuously differentiable on [0,1] .

Here, (A6)–(A10)are very common in the functional linear regression models.^20,21^ . (A6) prevents the spacing between eigenvalues from being too small to identify β(t) and (A7) ensures that β(t) is sufficiently smooth relative to the covariance function. (A11) ensures that the varying coefficient function is identifiable. (A12) to (A13) guarantee that the parameter $\alpha_{k}(u)$ s sufficiently smooth and can be approximated by spline functions, thereby ensuring that its estimation can achieve the optimal convergence rate. (A14) represents the smoothness condition of the varying coefficient function, describing the requirements for the optimal convergence rate that can be achieved in estimating the varying coefficient function.^22^

**Theorem 1:** Under conditions A(1)–A(14), $m∼n^{1/(a+2b)}$ and $k_{n}∼n^{1/(1+2r)}$ , we have

$$\begin{array}{rl} & {\left\|\hat{\beta }(\cdot )-\beta_{0}(\cdot )\right\|}^{2}=O_{p}\left(n^{-\frac{2b-1}{a+2b}}\right)+O_{p}\left(n^{-\frac{2b}{a+2b}+\frac{1}{2r+1}}\right),\;\text{and} \end{array}$$

$$\begin{array}{rl} & {\left\|\hat{\alpha }(\cdot )-\alpha_{0}(\cdot )\right\|}^{2}=O_{p}\left(n^{-\frac{a+2b-1}{a+2b}}\right)+O_{p}\left(n^{-2r/(2r+1)}\right). \end{array}$$

## 5. Simulation studies

### 5.1. Model setting

In this section, simulation studies are conducted to study the performance of the FVC-Cox model. First, we assume that the data are coming from the following model:

$$h(t|X,Z,V)=h_{0}(t)\exp\;\left(V\theta +Z\alpha (U)+\int_{0}^{1}X(t)\beta (t)dt\right),$$

where $h_{0}(t)=1$ , $\theta =(\theta_{1},\theta_{2},\theta_{3}{)}^{T}=(1,0.15,0.35{)}^{T}$ , and $Z\alpha (U)=\alpha_{1}(U)Z_{1}+\alpha_{2}(U)Z_{2}$ with $\alpha_{1}(U)=2\cos\;(2\pi U)$ and $\alpha_{2}(U)=-2+(3-U{)}^{3}/5$ . For the functional data, let $\beta (t)=\sqrt{2}sin(\pi t/2)+3\sqrt{2}sin(3\pi t/2)$ . For the random function, we set $X(t)=\sum_{i=1}^{\infty }\xi_{i}\phi_{i}(t)$ with $\xi_{i}$ following $N(0,\lambda_{i})$ , $\lambda_{i}=((i-0.5)\pi {)}^{-2}$ and $\phi_{j}(t)=\sqrt{2}sin((j-0.5)\pi t)$ . According to (3), it can be seen that when performing parameter estimation on the functional part, it is hard to directly obtain the result of the unknown β(t) through parameter estimation. However, after decomposing β(t) , since the coefficient $(1,3{)}^{T}$ in the expression $\beta (t)=\sqrt{2}sin(\pi t/2)+3\sqrt{2}sin(3\pi t/2)$ can be estimated, the subsequent numerical comparison of functional parameter estimation in the simulation will be mainly focused on the parameters $(1,3{)}^{T}$ .

### 5.2. Analysis of simulation results

In this section, we consider scenarios with sample sizes of 500, 1000, and 2000, and simultaneously evaluates the proposed model under censoring rates of 0.1, 0.3, and 0.5. For all nine scenarios, we assess the performance of the model and estimators based on the root of average squared error (RASE) and integrated bias (IBIAS), which are defined as follows:

$$\begin{array}{rl} \mathrm{RASE}(\hat{\beta }) & ={\left(\frac{1}{n}\sum_{i=1}^{n}(\hat{\theta }-\theta {)}^{2}\right)}^{1/2}, \end{array}$$

$$\begin{array}{rl} \mathrm{IBIAS}(\hat{\alpha (\cdot )}) & =\int_{0}^{1}(E(\hat{\alpha (u)})-\alpha (u){)}^{2}du,\;\text{and} \end{array}$$

$$\begin{array}{rl} \mathrm{IBIAS}(\hat{\beta (\cdot )}) & =\int_{0}^{1}(E(\hat{\beta (t)})-\beta (t){)}^{2}dt. \end{array}$$

**Figure 1.**
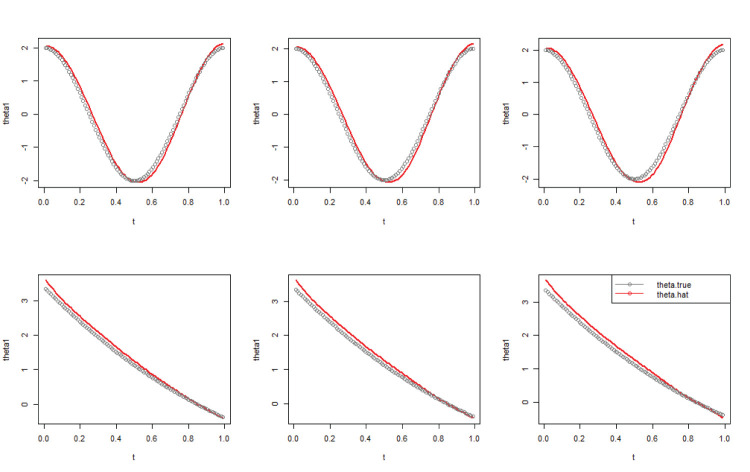
The true and estimated values of the varying coefficient part at three different quantiles, sample size is 500.

Table 1 shows the corresponding simulation results for the nine scenarios. As expected, all RASEs and IBIASs increase with censoring rate and decrease with sample size. Figure 1 plots the true and estimated values of the varying coefficient part at three different quantiles when the sample size is 500. According to Figure 1, there are insignificant discrepancies between the true values and the estimated values of the varying coefficients obtained from our proposed method. These results suggest that our model and estimation method are highly effective for estimating Cox models with varying coefficients.

**Table 1.** RASE and IBIAS for sample sizes of 500, 1000, and 2000 at censoring rates of 0.1, 0.3, and 0.5 (SZ: sample size; CR: censoring rate).

SZ	CR	$\theta_{1}$	$\theta_{2}$	$\theta_{3}$	$\beta_{1}$	$\beta_{2}$	$\alpha_{1}$	$\alpha_{2}$
	0.1	0.036	0.004	0.010	0.037	0.092	0.028	0.025
500	0.3	0.040	0.000	0.010	0.040	0.094	0.030	0.028
	0.5	0.048	0.000	0.011	0.053	0.111	0.033	0.031
	0.1	0.014	0.003	0.008	0.018	0.043	0.005	0.013
1000	0.3	0.018	0.003	0.010	0.021	0.056	0.006	0.014
	0.5	0.022	0.005	0.010	0.026	0.063	0.006	0.014
	0.1	0.008	0.002	0.002	0.008	0.017	0.007	0.012
2000	0.3	0.009	0.001	0.002	0.007	0.027	0.007	0.012
	0.5	0.009	0.000	0.002	0.009	0.033	0.006	0.012

### 5.3. Comparison between varying coefficient and fixed coefficient functional Cox models

When the data is under the same model setting, a comparison can be made between the results of simulations with and without varying coefficients. To save space, only the results under fixed coefficient settings with censoring rates of 0.1, 0.3, and 0.5, and sample sizes of 500, 1000, and 2000 are presented in Table 2.

**Table 2.** RASE and IBIAS for fixed coefficient settings at censoring rates of 0.1, 0.3, and 0.5, and sample sizes of 500, 1000, and 2000 (SZ: sample size; CR: censoring rate).

SZ	CR	$\theta_{1}$	$\theta_{2}$	$\theta_{3}$	$\beta_{1}$	$\beta_{2}$	$\alpha_{1}$	$\alpha_{2}$
	0.1	0.551	0.084	0.190	0.549	1.667	2.929	2.711
500	0.3	0.537	0.080	0.185	1.021	3.000	2.920	2.693
	0.5	0.536	0.078	0.184	1.020	3.000	2.874	2.758
	0.1	0.560	0.084	0.193	0.562	1.686	2.802	2.745
1000	0.3	0.545	0.081	0.189	0.549	1.636	2.831	2.711
	0.5	0.545	0.081	0.189	0.550	1.637	2.822	2.804
	0.1	0.560	0.081	0.200	0.559	1.692	2.801	2.758
2000	0.3	0.544	0.078	0.195	0.545	1.648	2.824	2.758
	0.5	0.544	0.079	0.195	0.545	1.644	2.800	2.751

According to Table 2, it can be seen that when the varying coefficient part of the model is replaced with fixed coefficients, there is a significant difference between the simulated results and the true results. Compared to Table 1, the RASEs and IBIASs in Table 2 become much larger. These results clearly suggest that for varying coefficient functional data, the original functional Cox model cannot provide a good fit and estimation of the data. The FVC-Cox model proposed in this paper, however, can not only encompass both functional covariate effects and varying coefficient effects but also perform parameter estimation more effectively than the functional Cox model.

## 6. Real data analysis

The data used in this paper were obtained from the ADNI database (https://adni.loni.usc.edu/). The primary objective of ADNI is to test whether a combination of serial magnetic resonance imaging (MRI) and other biomarkers can be used to measure the progression of mild cognitive impairment (MCI) and early Alzheimer’s disease (AD) since early and more accurate diagnosis of AD is considered an important therapeutic measure. This is the goal of researchers because therapeutic interventions are more likely to be beneficial during the early stages of the disease. At the same time, identifying sensitive and specific markers of early AD progression aims to help researchers and clinicians develop new treatments, monitor their effectiveness, and reduce the time and cost of clinical trials.

The hippocampus is one of the key brain regions affected by AD. The data set contains clinical and imaging measurements from 373 MCI individuals in ADNI, using them to predict the time from MCI conversion to AD. Among the 373 MCI individuals, 161 MCI individuals progressed to AD before the study was completed, while the remaining 212 individuals did not convert to AD by the end of the study. Therefore, the time from MCI to AD conversion can be regarded as time-to-event data,and the outcomes are interval censored.

In this paper, we use the proposed FVC-Cox model to fit the ADNI dataset. Here, the covariates include gender (1 = male; 0 = female), handedness (1 = right-handed; 2 = left-handed), whether widowed (0 = no; 1 = yes), whether divorced (0 = no; 1 = yes), whether married (0 = no; 1 = yes), years of education, whether retired (1 = yes; 0 = no), age, whether the first allele is genotype 3 (0 = no; 1 = yes), whether the first allele is genotype 4 (0 = no; 1 = yes), whether the second allele is genotype 3 (0 = no; 1 = yes), and ADAS-Cog score. This results in a design matrix Z with dimensions (n, p) = (373, 12). For the functional predictor variables, we used the hippocampal radial distances at 2000 surface points on the left and right hippocampal surfaces as functional data. The radial distance, defined as the distance between the medial core of the hippocampus and the corresponding vertex, is a summary statistic of hippocampal shape and size.

**Figure 2.**
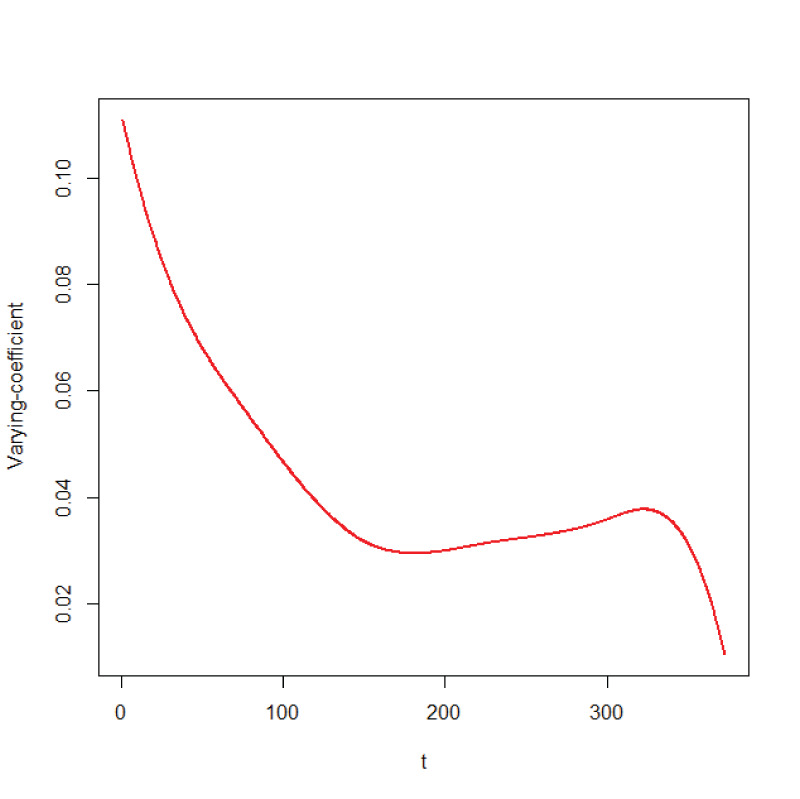
The variation of the varying coefficients α(age) with age.

**Figure 3.**
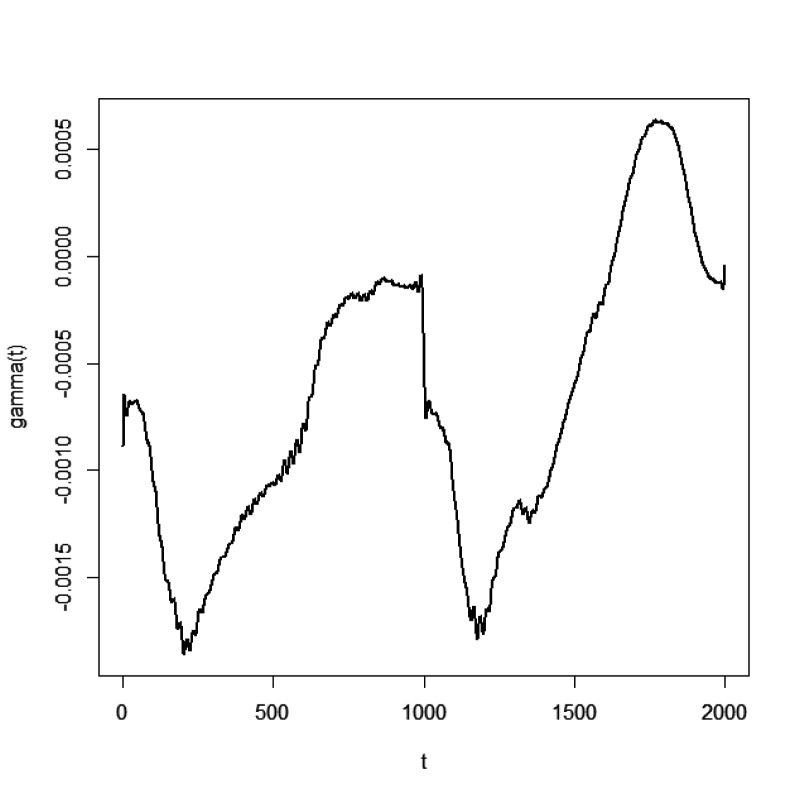
Functional coefficients β(t) in ADNI data.

Here, the interaction between ADAS-Cog score and age was considered, meaning that the coefficient of ADAS-Cog score is a function of age, denoted as α(U)=α(age) . First, principal components are estimated by applying FPCA to the hippocampal radial distances. Next, the varying coefficient part is expanded using B-spline methods. Finally, parameters are estimated using FVC-Cox model and the proposed parameter estimation method. Based on the AIC criterion, the first three principal components are selected and these three principal components explain a total of 76.4% of the total variance. By utilizing the proposed FVC-Cox model and its estimation method, we can obtain the estimated values for the linear part θ (see Table 3) and the estimated values for the varying coefficient part α (age) (see Figure 2). For the functional coefficient β(t) , the graph of this coefficient can be derived using the formula $\hat{\beta }(t)=\sum_{j=1}^{\infty }\eta_{j}{\hat{\phi }}_{j}(t)$ , as shown in Figure 3.

**Table 3.** Estimated values of the linear component θ .

	$\theta_{4}$	$\theta_{5}$	$\theta_{6}$	$\theta_{7}$	$\theta_{8}$	$\theta_{9}$	$\theta_{10}$
Estimate	0.294	−0.127	−0.210	−0.153	0.139	0.015	−0.016
Standard error	0.187	0.327	0.302	0.365	0.765	0.027	0.216
	$\beta_{11}$	$\theta_{12}$	$\theta_{13}$	$\theta_{14}$
Estimate	−0.005	0.023	−0.077	−0.647			
Standard error	0.013	0.417	0.460	0.193			

From Figure 2, the analysis results show that the coefficient of the ADAS-Cog score is a non-linear curve. For data with varying coefficients, using only fixed coefficients Cox analysis can lead to biased coefficient estimates and other issues. Therefore, the FVC-Cox model proposed in this paper is appropriate. Table 3 reports the parameter estimates and their corresponding standard errors. It can be seen that ADAS-Cog score is a significant factor, which is consistent with the conclusions from Kong et al.^18^ This result suggests that the ADAS-Cog score provides a good predictive power for the conversion from MCI to AD.

## 7. Conclusion

As data become more complex, the demand for model flexibility becomes higher. The functional Cox model incorporates functional data into the Cox model, increasing model flexibility and expanding the scope of application for functional data. Building on the functional Cox model, we further incorporate varying coefficients, which further enhances the flexibility of the survival model. Analysis of simulated data shows that this model performs better in scenarios where both functional data and varying coefficients exist at the same time than the functional Cox model. Our proposed model and estimation method also perform well in the real data analysis.

## Appendix

**Appendix**

Proof Proof of Theorem 1 For convenience, we define $V=(V_{1},\ldots ,V_{n}{)}^{T}$ and $\omega =(\omega_{1},\ldots ,\omega_{n}{)}^{T}$ are quasidesign matrix for slope function and varying-coefficient function component, respectively. Let $\eta_{0}=(\eta_{0,1},\ldots ,\eta_{0,m}{)}^{T}$ be coefficient vector for true slope function $\beta_{0}(t)$ . $\gamma_{0}=(\gamma_{01}^{T},\ldots ,\gamma_{0p}^{T}{)}^{T}$ be the true coefficients for the spline basis, $B=\mathrm{diag}\;(f_{1}(0),\ldots ,f_{n}(0)),P=\omega (\omega^{T}B\omega {)}^{-1}\omega^{T}B,V^{*}=(I-P)V=(V_{1}^{*},\ldots ,V_{n}^{*}{)}^{T}$ , $H_{n}{}^{2}=k_{n}\omega^{T}B\omega$ and $\Lambda_{n}=V^{*T}BV^{*}$ . We also let

θ(ηγ)=(θ1θ2)=(Λn12(η−η0)kn−12Hn(γ−γ0)+knHn−1ωTBV(η−η0))

and

$$\hat{\theta }=\theta (\hat{\eta },\hat{\gamma }{)}^{T}={\left({\hat{\theta }}_{1}^{T},{\hat{\theta }}_{2}^{T}\right)}^{T}$$

Observe that

$$\begin{array}{rl} {\left\|\hat{\beta }(t)-\beta_{0}(t)\right\|}^{2} & ={\left\|\sum_{j=1}^{m}{\hat{\eta }}_{j}{\hat{\phi }}_{j}(t)-\sum_{j=1}^{\infty }\eta_{0j}\phi_{j}(t)\right\|}^{2}\\ & \leq 2{\left\|\sum_{j=1}^{m}{\hat{\eta }}_{j}{\hat{\phi }}_{j}(t)-\sum_{j=1}^{m}\eta_{0j}\phi_{j}(t)\right\|}^{2}+2{\left\|\sum_{j=m+1}^{\infty }\eta_{0j}\phi_{j}(t)\right\|}^{2}\\ & \leq 4{\left\|\sum_{j=1}^{m}({\hat{\eta }}_{j}-\eta_{0j}){\hat{\phi }}_{j}(t)\right\|}^{2}+4{\left\|\sum_{j=1}^{m}\eta_{0j}({\hat{\phi }}_{j}(t)-\phi_{j}(t))\right\|}^{2}\\ & \;+2\sum_{j=m+1}^{\infty }\eta_{0j}^{2}\\ & =4A_{1}+4A_{2}+2A_{3} \end{array}$$

where,

$$\begin{array}{rl} A_{1} & ={\left\|\sum_{j=1}^{m}({\hat{\eta }}_{j}-\eta_{0j}){\hat{\phi }}_{j}(t)\right\|}^{2} \end{array}$$

$$\begin{array}{rl} A_{2} & ={\left\|\sum_{j=1}^{m}\eta_{0j}({\hat{\phi }}_{j}(t)-\phi_{j}(t))\right\|}^{2} \end{array}$$

$$\begin{array}{rl} A_{3} & =\sum_{j=m+1}^{\infty }\eta_{0j}^{2} \end{array}$$

Invoking the orthogonality of ${\hat{\phi }}_{j}$ ,we have:

$$\begin{array}{rl} A_{1} & ={\left\|\sum_{j=1}^{m}({\hat{\eta }}_{j}-\eta_{0j}){\hat{\phi }}_{j}(t)\right\|}^{2}\leq \sum_{j=1}^{m}{\left|{\hat{\eta }}_{j}-\eta_{0j}\right|}^{2}={\left\|\hat{\eta }-\eta_{0}\right\|}^{2}\\ & =O_{p}\left(a_{n}^{2}\right)=O_{p}\left(n^{-\frac{2b-1}{a+2b}}\right)+O_{p}\left(k_{n}n^{-\frac{2b}{a+2b}}\right)\\ A_{2} & ={\left\|\sum_{j=1}^{m}\eta_{0j}\left({\hat{\phi }}_{j}-\phi_{j}\right)\right\|}^{2}\leq m\sum_{j=1}^{m}{\left\|{\hat{\phi }}_{j}-\phi_{j}\right\|}^{2}\eta_{0j}^{2}\leq \frac{m}{n}\sum_{j=1}^{m}j^{2}\eta_{0j}^{2}\\ & =O_{p}\left(n^{-1}m\sum_{j=1}^{m}j^{2-2b}\right)=O_{p}\left(n^{-1}m\right)=o_{p}\left(n^{-\frac{2b-1}{a+2b}}\right)\\ A_{3} & =\sum_{j=m+1}^{\infty }\eta_{0j}^{2}\leq K\sum_{j=m+1}^{\infty }j^{-2b}=O\left(n^{-\frac{2b-1}{a+2b}}\right) \end{array}$$

Then ,we can have:

$${\left\|\hat{\beta }(\cdot )-\beta_{0}(\cdot )\right\|}^{2}=4A_{1}+4A_{2}+2A_{3}=O_{p}\left(n^{-\frac{2b-1}{a+2b}}\right)+O_{p}\left(n^{-\frac{2b}{a+2b}+\frac{1}{2r+1}}\right)$$

By and Cauchy–Schwarz inequality and the definition of $\hat{\theta }$ ,we have:

$$\begin{array}{rl} {\left\|\hat{\gamma }-\gamma_{0}\right\|}^{2} & ={\left\|\sqrt{k_{n}}H_{n}^{-1}{\hat{\theta }}_{2}-k_{n}H_{n}{}^{-2}\omega^{T}BV\left(\hat{\eta }-\eta_{0}\right)\right\|}^{2}\\ & \leq {\left\|\sqrt{k_{n}}H_{n}^{-1}{\hat{\theta }}_{2}\right\|}^{2}+{\left\|k_{n}H_{n}^{-2}\omega^{T}BV\left(\hat{\eta }-\eta_{0}\right)\right\|}^{2}\\ & =k_{n}{\hat{\theta }}_{2}^{T}H_{n}^{-2}{\hat{\theta }}_{2}+k_{n}^{2}{\left(\hat{\eta }-\eta_{0}\right)}^{T}V^{T}B\omega H_{n}^{-4}\omega^{T}BV\left(\hat{\eta }-\eta_{0}\right)\\ & =k_{n}{\hat{\theta }}_{2}^{T}H_{n}^{-2}{\hat{\theta }}_{2}+k_{n}^{2}{\left(\hat{\eta }-\eta_{0}\right)}^{T}V^{T}B\omega H_{n}^{-2}{\left(k_{n}\omega^{T}B\omega \right)}^{-1}\omega^{T}BV\left(\hat{\eta }-\eta_{0}\right)\\ & =O_{p}\left(\frac{k_{n}\left(k_{n}+m\right)}{n}\right)\\ & \;+k_{n}\left(O_{p}\left(n^{-\frac{2b-1}{a+2b}}\right)+O_{p}\left(k_{n}n^{-\frac{2b}{a+2b}}\right)\right)O_{p}\left(\frac{1}{n}\right)O_{p}\left(n{\hat{\lambda }}_{m}\right)\\ & =O_{p}\left(\frac{k_{n}\left(k_{n}+m\right)}{n}\right). \end{array}$$

Therefore, by Hölder inequality and the above equation, for l=1,…,p ,we have

$$\begin{array}{rl} {\left\|{\hat{\alpha }}_{l}(u)-\alpha_{0l}(u)\right\|}^{2} & ={\left\|\omega^{T}(u){\hat{\gamma }}_{l}-\alpha_{0l}(u)\right\|}^{2}\\ & \leq 2{\left\|\omega^{T}(u)\left({\hat{\gamma }}_{l}-\gamma_{0l}\right)\right\|}^{2}+2{\left\|\alpha_{0l}(u)-\omega^{T}(u)\gamma_{0I}\right\|}^{2}\\ & =O\left(\frac{1}{k_{n}}\right){\left\|{\hat{\gamma }}_{l}-\gamma_{0l}\right\|}^{2}+O_{p}\left(k_{n}^{-2r}\right)\\ & =O\left(\frac{1}{k_{n}}\right)O_{p}\left(\frac{k_{n}\left(k_{n}+m\right)}{n}\right)+O_{p}\left(n^{-2r/(2r+1)}\right)\\ & =O_{p}\left(n^{-(a+2b-1)/(a+2b)}\right)+O_{p}\left(n^{-2r/(2r+1)}\right) \end{array}$$

Thus, this completes the proof of Theorem 1.
